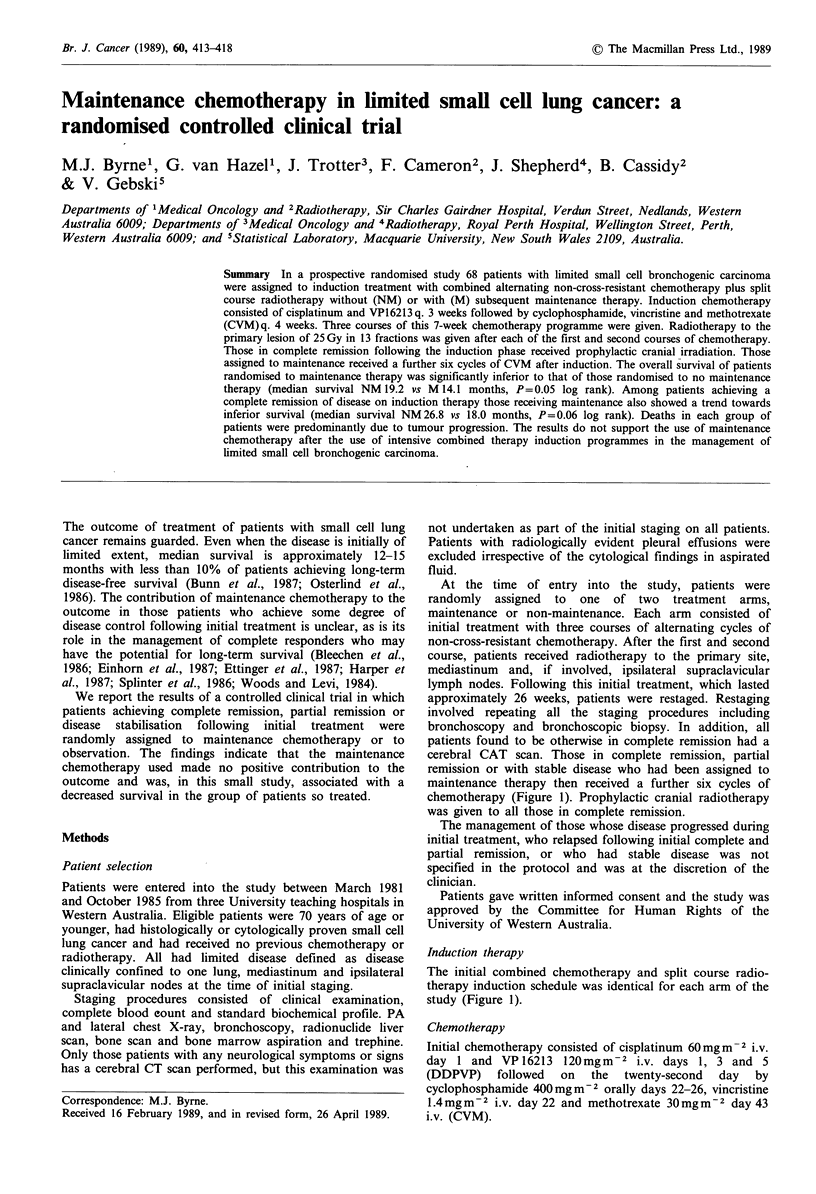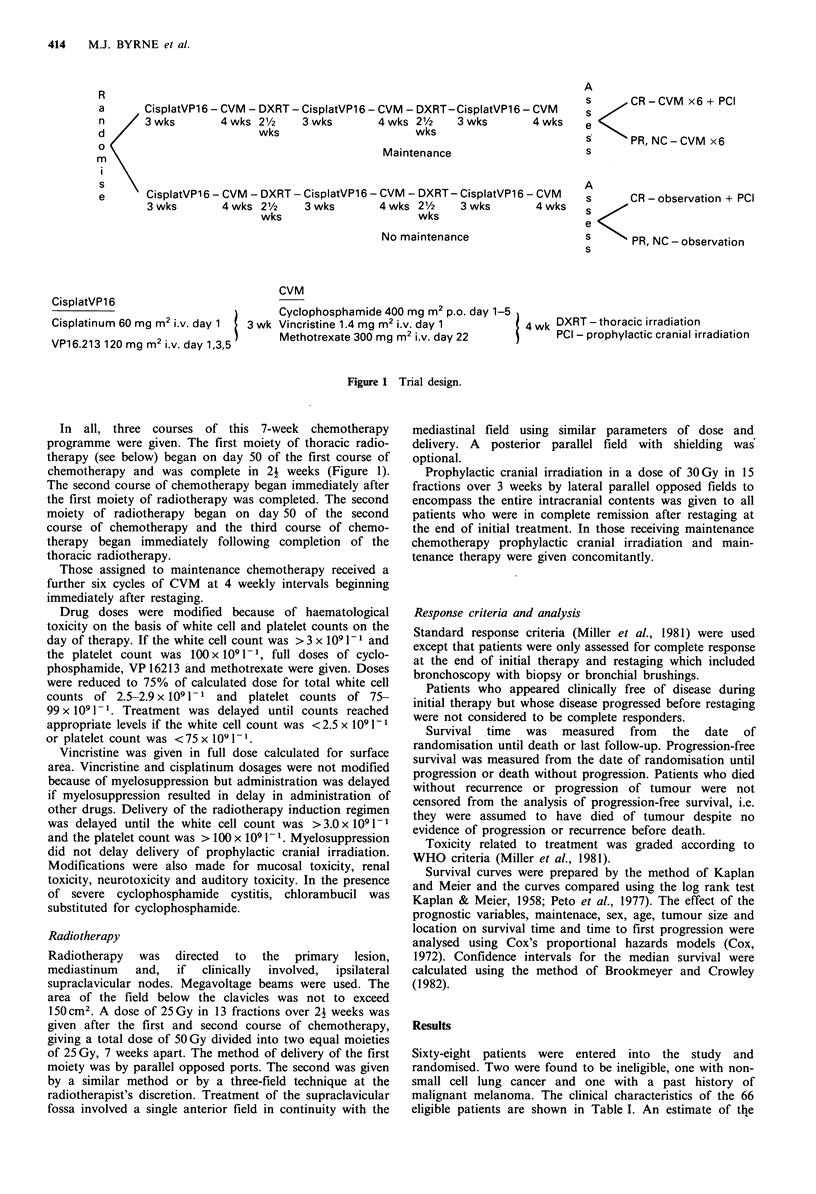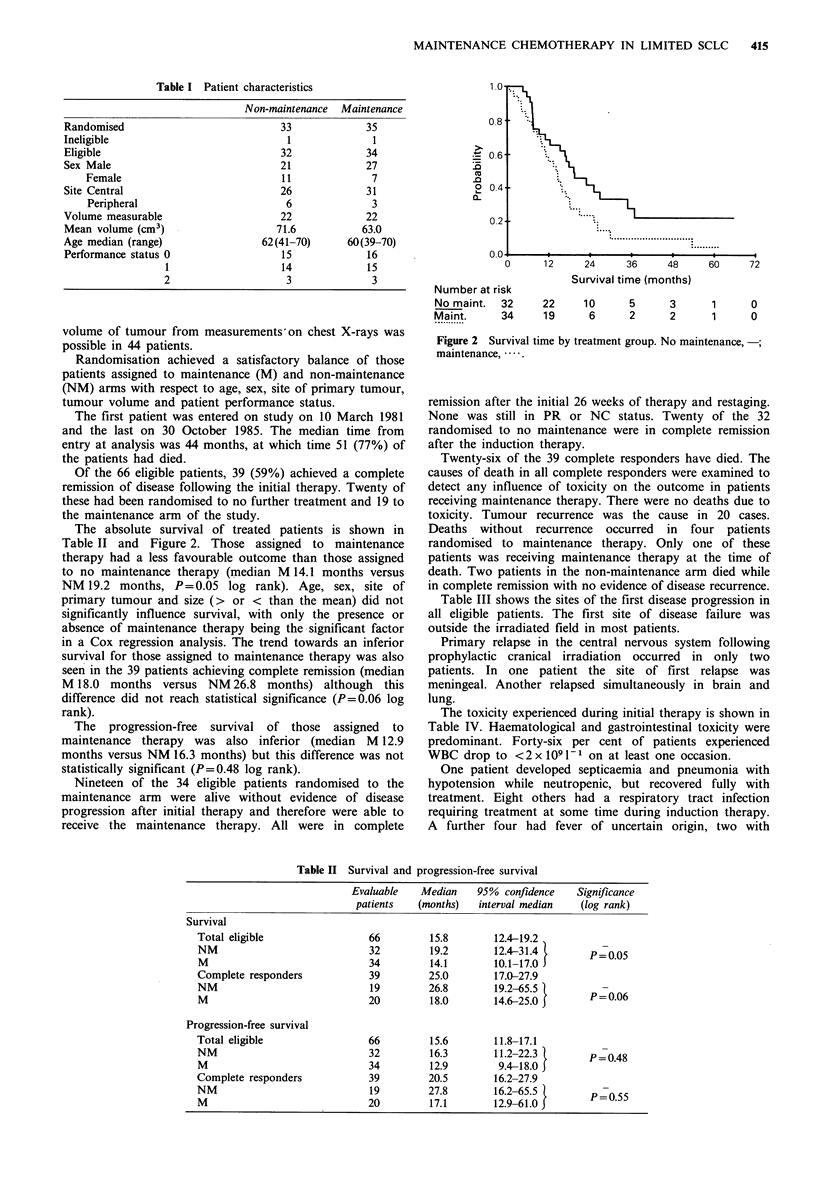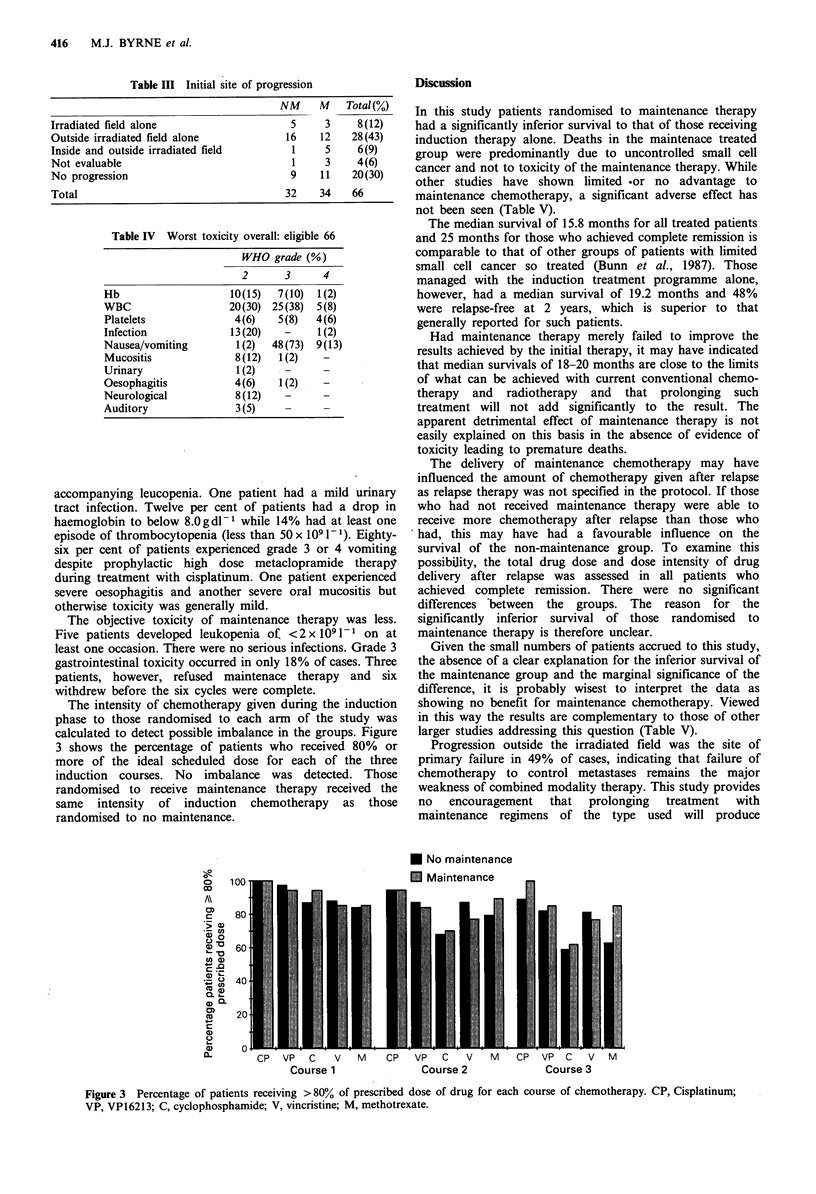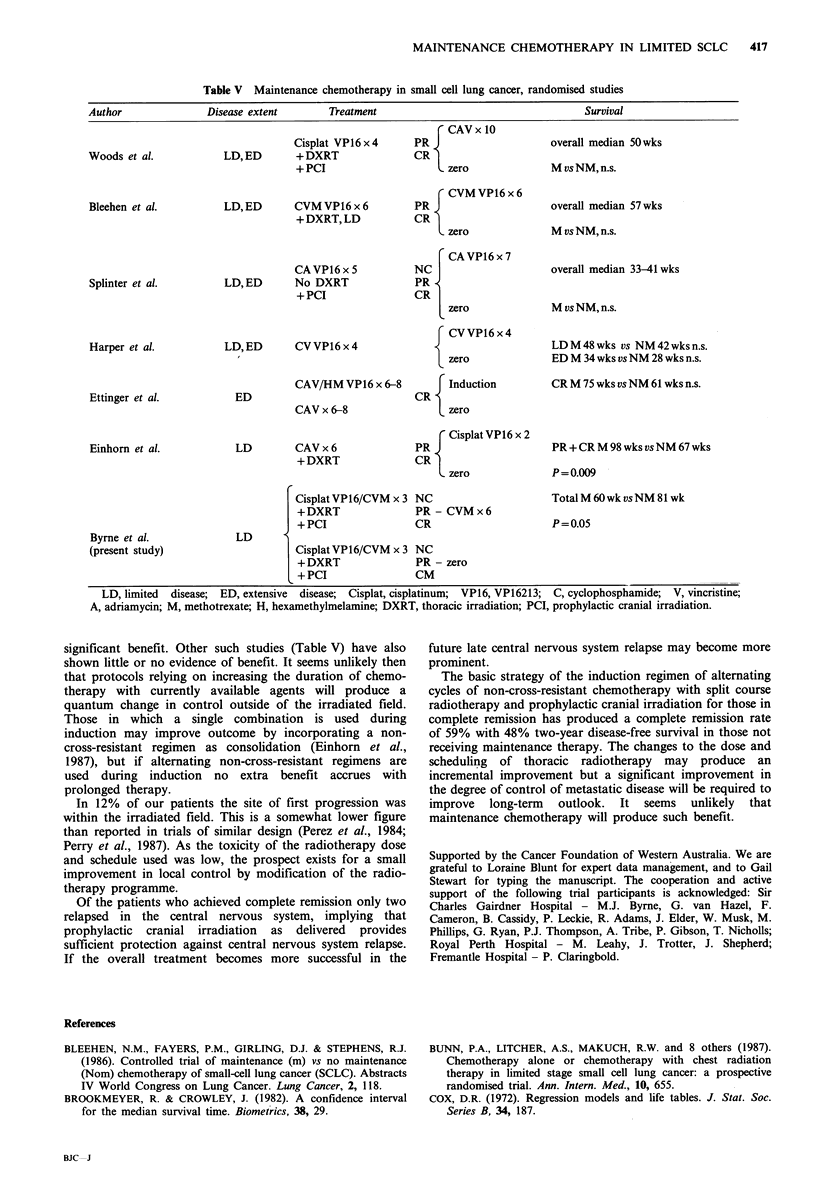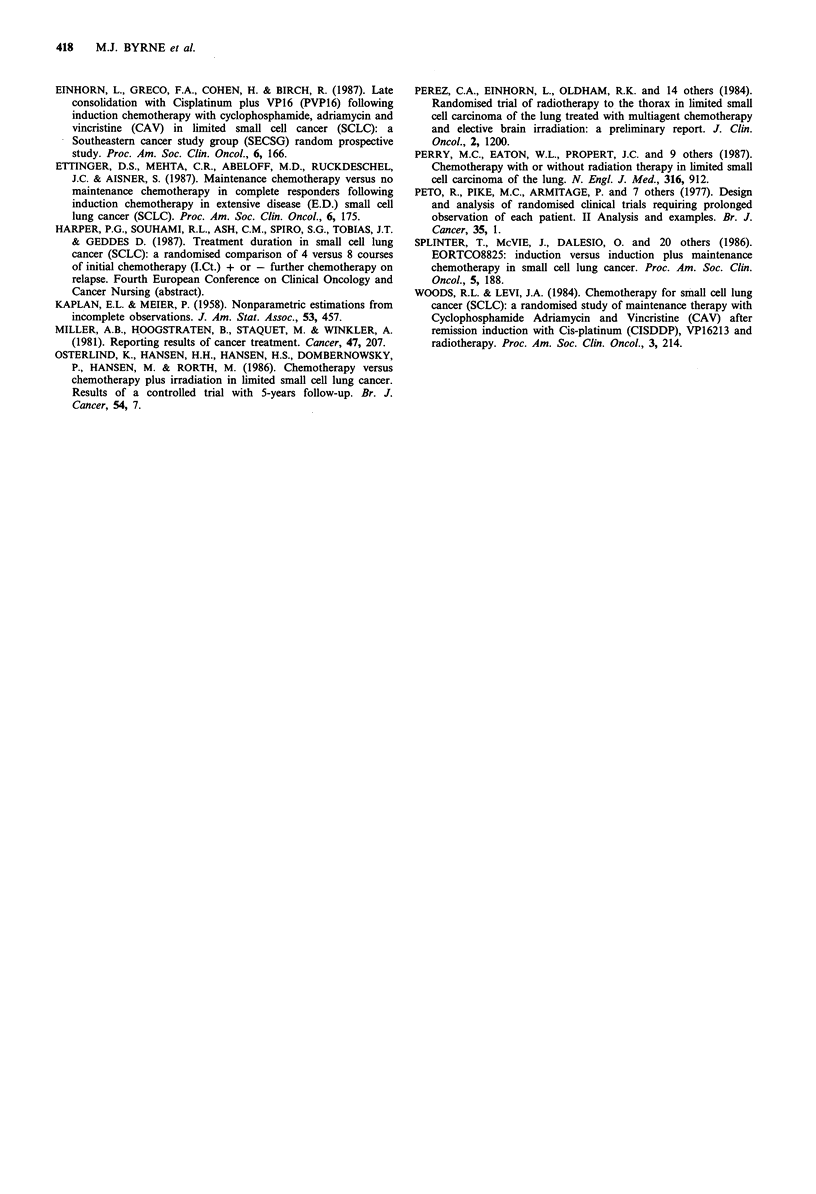# Maintenance chemotherapy in limited small cell lung cancer: a randomised controlled clinical trial.

**DOI:** 10.1038/bjc.1989.296

**Published:** 1989-09

**Authors:** M. J. Byrne, G. van Hazel, J. Trotter, F. Cameron, J. Shepherd, B. Cassidy, V. Gebski

**Affiliations:** Department of Medical Oncology, Sir Charles Gairdner Hospital, Nedlands, Western Australia.

## Abstract

In a prospective randomised study 68 patients with limited small cell bronchogenic carcinoma were assigned to induction treatment with combined alternating non-cross-resistant chemotherapy plus split course radiotherapy without (NM) or with (M) subsequent maintenance therapy. Induction chemotherapy consisted of cisplatinum and VP16213q. 3 weeks followed by cyclophosphamide, vincristine and methotrexate (CVM)q. 4 weeks. Three courses of this 7-week chemotherapy programme were given. Radiotherapy to the primary lesion of 25 Gy in 13 fractions was given after each of the first and second courses of chemotherapy. Those in complete remission following the induction phase received prophylactic cranial irradiation. Those assigned to maintenance received a further six cycles of CVM after induction. The overall survival of patients randomised to maintenance therapy was significantly inferior to that of those randomised to no maintenance therapy (median survival NM 19.2 vs M 14.1 months, P = 0.05 log rank). Among patients achieving a complete remission of disease on induction therapy those receiving maintenance also showed a trend towards inferior survival (median survival NM 26.8 vs 18.0 months, P = 0.06 log rank). Deaths in each group of patients were predominantly due to tumour progression. The results do not support the use of maintenance chemotherapy after the use of intensive combined therapy induction programmes in the management of limited small cell bronchogenic carcinoma.


Br. J. Cancer (1989), 60, 413–418 © The Macmillan Press Ltd., 1989

### Maintenance chemotherapy in limited small cell lung cancer: a randomised controlled clinical trial

M.J. Byrne[1], G. van Hazel[1], J. Trotter[3], F. Cameron[2], J. Shepherd[4], B. Cassidy[2] & V. Gebski[5]

*Departments of [1]Medical Oncology and [2]Radiotherapy, Sir Charles Gairdner Hospital, Verdun Street, Nedlands, Western Australia 6009; Departments of [3]Medical Oncology and [4]Radiotherapy, Royal Perth Hospital, Wellington Street, Perth, Western Australia 6009; and [5]Statistical Laboratory, Macquarie University, New South Wales 2109, Australia.*

**Summary** In a prospective randomised study 68 patients with limited small cell bronchogenic carcinoma were assigned to induction treatment with combined alternating non-cross-resistant chemotherapy plus split course radiotherapy without (NM) or with (M) subsequent maintenance therapy. Induction chemotherapy consisted of cisplatinum and VP16213 q. 3 weeks followed by cyclophosphamide, vincristine and methotrexate (CVM) q. 4 weeks. Three courses of this 7-week chemotherapy programme were given. Radiotherapy to the primary lesion of 25 Gy in 13 fractions was given after each of the first and second courses of chemotherapy. Those in complete remission following the induction phase received prophylactic cranial irradiation. Those assigned to maintenance received a further six cycles of CVM after induction. The overall survival of patients randomised to maintenance therapy was significantly inferior to that of those randomised to no maintenance therapy (median survival NM 19.2 *vs* M 14.1 months, $P=0.05$ log rank). Among patients achieving a complete remission of disease on induction therapy those receiving maintenance also showed a trend towards inferior survival (median survival NM 26.8 *vs* 18.0 months, $P=0.06$ log rank). Deaths in each group of patients were predominantly due to tumour progression. The results do not support the use of maintenance chemotherapy after the use of intensive combined therapy induction programmes in the management of limited small cell bronchogenic carcinoma.

The outcome of treatment of patients with small cell lung cancer remains guarded. Even when the disease is initially of limited extent, median survival is approximately 12–15 months with less than 10% of patients achieving long-term disease-free survival (Bunn *et al.*, 1987; Osterlind *et al.*, 1986). The contribution of maintenance chemotherapy to the outcome in those patients who achieve some degree of disease control following initial treatment is unclear, as is its role in the management of complete responders who may have the potential for long-term survival (Bleechen *et al.*, 1986; Einhorn *et al.*, 1987; Ettinger *et al.*, 1987; Harper *et al.*, 1987; Splinter *et al.*, 1986; Woods and Levi, 1984).

We report the results of a controlled clinical trial in which patients achieving complete remission, partial remission or disease stabilisation following initial treatment were randomly assigned to maintenance chemotherapy or to observation. The findings indicate that the maintenance chemotherapy used made no positive contribution to the outcome and was, in this small study, associated with a decreased survival in the group of patients so treated.

### Methods

### *Patient selection*

Patients were entered into the study between March 1981 and October 1985 from three University teaching hospitals in Western Australia. Eligible patients were 70 years of age or younger, had histologically or cytologically proven small cell lung cancer and had received no previous chemotherapy or radiotherapy. All had limited disease defined as disease clinically confined to one lung, mediastinum and ipsilateral supraclavicular nodes at the time of initial staging.

Staging procedures consisted of clinical examination, complete blood count and standard biochemical profile. PA and lateral chest X-ray, bronchoscopy, radionuclide liver scan, bone scan and bone marrow aspiration and trephine. Only those patients with any neurological symptoms or signs has a cerebral CT scan performed, but this examination was not undertaken as part of the initial staging on all patients. Patients with radiologically evident pleural effusions were excluded irrespective of the cytological findings in aspirated fluid.

At the time of entry into the study, patients were randomly assigned to one of two treatment arms, maintenance or non-maintenance. Each arm consisted of initial treatment with three courses of alternating cycles of non-cross-resistant chemotherapy. After the first and second course, patients received radiotherapy to the primary site, mediastinum and, if involved, ipsilateral supraclavicular lymph nodes. Following this initial treatment, which lasted approximately 26 weeks, patients were restaged. Restaging involved repeating all the staging procedures including bronchoscopy and bronchoscopic biopsy. In addition, all patients found to be otherwise in complete remission had a cerebral CAT scan. Those in complete remission, partial remission or with stable disease who had been assigned to maintenance therapy then received a further six cycles of chemotherapy (Figure 1). Prophylactic cranial radiotherapy was given to all those in complete remission.

The management of those whose disease progressed during initial treatment, who relapsed following initial complete and partial remission, or who had stable disease was not specified in the protocol and was at the discretion of the clinician.

Patients gave written informed consent and the study was approved by the Committee for Human Rights of the University of Western Australia.

### *Induction therapy*

The initial combined chemotherapy and split course radiotherapy induction schedule was identical for each arm of the study (Figure 1).

### *Chemotherapy*

Initial chemotherapy consisted of cisplatinum 60 mg m$^{-2}$ i.v. day 1 and VP16213 120 mg m$^{-2}$ i.v. days 1, 3 and 5 (DDPVP) followed on the twenty-second day by cyclophosphamide 400 mg m$^{-2}$ orally days 22–26, vincristine 1.4 mg m$^{-2}$ i.v. day 22 and methotrexate 30 mg m$^{-2}$ day 43 i.v. (CVM).

Correspondence: M.J. Byrne.
Received 16 February 1989, and in revised form, 26 April 1989.

**Figure 1** Trial design.

In all, three courses of this 7-week chemotherapy programme were given. The first moiety of thoracic radiotherapy (see below) began on day 50 of the first course of chemotherapy and was complete in 2½ weeks (Figure 1). The second course of chemotherapy began immediately after the first moiety of radiotherapy was completed. The second moiety of radiotherapy began on day 50 of the second course of chemotherapy and the third course of chemotherapy began immediately following completion of the thoracic radiotherapy.

Those assigned to maintenance chemotherapy received a further six cycles of CVM at 4 weekly intervals beginning immediately after restaging.

Drug doses were modified because of haematological toxicity on the basis of white cell and platelet counts on the day of therapy. If the white cell count was $>3\times10^9 l^{-1}$ and the platelet count was $100\times10^9 l^{-1}$, full doses of cyclophosphamide, VP 16213 and methotrexate were given. Doses were reduced to 75% of calculated dose for total white cell counts of $2.5$–$2.9\times10^9 l^{-1}$ and platelet counts of 75–$99\times10^9 l^{-1}$. Treatment was delayed until counts reached appropriate levels if the white cell count was $<2.5\times10^9 l^{-1}$ or platelet count was $<75\times10^9 l^{-1}$.

Vincristine was given in full dose calculated for surface area. Vincristine and cisplatinum dosages were not modified because of myelosuppression but administration was delayed if myelosuppression resulted in delay in administration of other drugs. Delivery of the radiotherapy induction regimen was delayed until the white cell count was $>3.0\times10^9 l^{-1}$ and the platelet count was $>100\times10^9 l^{-1}$. Myelosuppression did not delay delivery of prophylactic cranial irradiation. Modifications were also made for mucosal toxicity, renal toxicity, neurotoxicity and auditory toxicity. In the presence of severe cyclophosphamide cystitis, chlorambucil was substituted for cyclophosphamide.

*Radiotherapy*

Radiotherapy was directed to the primary lesion, mediastinum and, if clinically involved, ipsilateral supraclavicular nodes. Megavoltage beams were used. The area of the field below the clavicles was not to exceed $150\,cm^2$. A dose of 25 Gy in 13 fractions over 2½ weeks was given after the first and second course of chemotherapy, giving a total dose of 50 Gy divided into two equal moieties of 25 Gy, 7 weeks apart. The method of delivery of the first moiety was by parallel opposed ports. The second was given by a similar method or by a three-field technique at the radiotherapist's discretion. Treatment of the supraclavicular fossa involved a single anterior field in continuity with the mediastinal field using similar parameters of dose and delivery. A posterior parallel field with shielding was optional.

Prophylactic cranial irradiation in a dose of 30 Gy in 15 fractions over 3 weeks by lateral parallel opposed fields to encompass the entire intracranial contents was given to all patients who were in complete remission after restaging at the end of initial treatment. In those receiving maintenance chemotherapy prophylactic cranial irradiation and maintenance therapy were given concomitantly.

*Response criteria and analysis*

Standard response criteria (Miller *et al.*, 1981) were used except that patients were only assessed for complete response at the end of initial therapy and restaging which included bronchoscopy with biopsy or bronchial brushings.

Patients who appeared clinically free of disease during initial therapy but whose disease progressed before restaging were not considered to be complete responders.

Survival time was measured from the date of randomisation until death or last follow-up. Progression-free survival was measured from the date of randomisation until progression or death without progression. Patients who died without recurrence or progression of tumour were not censored from the analysis of progression-free survival, i.e. they were assumed to have died of tumour despite no evidence of progression or recurrence before death.

Toxicity related to treatment was graded according to WHO criteria (Miller *et al.*, 1981).

Survival curves were prepared by the method of Kaplan and Meier and the curves compared using the log rank test Kaplan & Meier, 1958; Peto *et al.*, 1977). The effect of the prognostic variables, maintenace, sex, age, tumour size and location on survival time and time to first progression were analysed using Cox's proportional hazards models (Cox, 1972). Confidence intervals for the median survival were calculated using the method of Brookmeyer and Crowley (1982).

### Results

Sixty-eight patients were entered into the study and randomised. Two were found to be ineligible, one with non-small cell lung cancer and one with a past history of malignant melanoma. The clinical characteristics of the 66 eligible patients are shown in Table I. An estimate of the

**Table I** Patient characteristics

| | *Non-maintenance* | *Maintenance* |
|---|---|---|
| Randomised | 33 | 35 |
| Ineligible | 1 | 1 |
| Eligible | 32 | 34 |
| Sex Male | 21 | 27 |
| Female | 11 | 7 |
| Site Central | 26 | 31 |
| Peripheral | 6 | 3 |
| Volume measurable | 22 | 22 |
| Mean volume ($cm^3$) | 71.6 | 63.0 |
| Age median (range) | 62 (41–70) | 60 (39–70) |
| Performance status 0 | 15 | 16 |
| 1 | 14 | 15 |
| 2 | 3 | 3 |

volume of tumour from measurements on chest X-rays was possible in 44 patients.

Randomisation achieved a satisfactory balance of those patients assigned to maintenance (M) and non-maintenance (NM) arms with respect to age, sex, site of primary tumour, tumour volume and patient performance status.

The first patient was entered on study on 10 March 1981 and the last on 30 October 1985. The median time from entry at analysis was 44 months, at which time 51 (77%) of the patients had died.

Of the 66 eligible patients, 39 (59%) achieved a complete remission of disease following the initial therapy. Twenty of these had been randomised to no further treatment and 19 to the maintenance arm of the study.

The absolute survival of treated patients is shown in Table II and Figure 2. Those assigned to maintenance therapy had a less favourable outcome than those assigned to no maintenance therapy (median M 14.1 months versus NM 19.2 months, $P=0.05$ log rank). Age, sex, site of primary tumour and size (> or < than the mean) did not significantly influence survival, with only the presence or absence of maintenance therapy being the significant factor in a Cox regression analysis. The trend towards an inferior survival for those assigned to maintenance therapy was also seen in the 39 patients achieving complete remission (median M 18.0 months versus NM 26.8 months) although this difference did not reach statistical significance ($P=0.06$ log rank).

The progression-free survival of those assigned to maintenance therapy was also inferior (median M 12.9 months versus NM 16.3 months) but this difference was not statistically significant ($P=0.48$ log rank).

Nineteen of the 34 eligible patients randomised to the maintenance arm were alive without evidence of disease progression after initial therapy and therefore were able to receive the maintenance therapy. All were in complete

Number at risk

| | 0 | 12 | 24 | 36 | 48 | 60 | 72 |
|---|---|---|---|---|---|---|---|
| No maint. | 32 | 22 | 10 | 5 | 3 | 1 | 0 |
| Maint. | 34 | 19 | 6 | 2 | 2 | 1 | 0 |

**Figure 2** Survival time by treatment group. No maintenance, —; maintenance, ····.

remission after the initial 26 weeks of therapy and restaging. None was still in PR or NC status. Twenty of the 32 randomised to no maintenance were in complete remission after the induction therapy.

Twenty-six of the 39 complete responders have died. The causes of death in all complete responders were examined to detect any influence of toxicity on the outcome in patients receiving maintenance therapy. There were no deaths due to toxicity. Tumour recurrence was the cause in 20 cases. Deaths without recurrence occurred in four patients randomised to maintenance therapy. Only one of these patients was receiving maintenance therapy at the time of death. Two patients in the non-maintenance arm died while in complete remission with no evidence of disease recurrence.

Table III shows the sites of the first disease progression in all eligible patients. The first site of disease failure was outside the irradiated field in most patients.

Primary relapse in the central nervous system following prophylactic cranical irradiation occurred in only two patients. In one patient the site of first relapse was meningeal. Another relapsed simultaneously in brain and lung.

The toxicity experienced during initial therapy is shown in Table IV. Haematological and gastrointestinal toxicity were predominant. Forty-six per cent of patients experienced WBC drop to $<2\times10^9 l^{-1}$ on at least one occasion.

One patient developed septicaemia and pneumonia with hypotension while neutropenic, but recovered fully with treatment. Eight others had a respiratory tract infection requiring treatment at some time during induction therapy. A further four had fever of uncertain origin, two with

**Table II** Survival and progression-free survival

| | *Evaluable patients* | *Median (months)* | *95% confidence interval median* | *Significance (log rank)* |
|---|---|---|---|---|
| Survival | | | | |
| Total eligible | 66 | 15.8 | 12.4–19.2 | |
| NM | 32 | 19.2 | 12.4–31.4 | $P=0.05$ |
| M | 34 | 14.1 | 10.1–17.0 | |
| Complete responders | 39 | 25.0 | 17.0–27.9 | |
| NM | 19 | 26.8 | 19.2–65.5 | $P=0.06$ |
| M | 20 | 18.0 | 14.6–25.0 | |
| Progression-free survival | | | | |
| Total eligible | 66 | 15.6 | 11.8–17.1 | |
| NM | 32 | 16.3 | 11.2–22.3 | $P=0.48$ |
| M | 34 | 12.9 | 9.4–18.0 | |
| Complete responders | 39 | 20.5 | 16.2–27.9 | |
| NM | 19 | 27.8 | 16.2–65.5 | $P=0.55$ |
| M | 20 | 17.1 | 12.9–61.0 | |

**Table III** Initial site of progression

| | NM | M | Total(%) |
|---|---|---|---|
| Irradiated field alone | 5 | 3 | 8(12) |
| Outside irradiated field alone | 16 | 12 | 28(43) |
| Inside and outside irradiated field | 1 | 5 | 6(9) |
| Not evaluable | 1 | 3 | 4(6) |
| No progression | 9 | 11 | 20(30) |
| Total | 32 | 34 | 66 |

**Table IV** Worst toxicity overall: eligible 66

| | WHO grade (%) | | |
|---|---|---|---|
| | 2 | 3 | 4 |
| Hb | 10(15) | 7(10) | 1(2) |
| WBC | 20(30) | 25(38) | 5(8) |
| Platelets | 4(6) | 5(8) | 4(6) |
| Infection | 13(20) | – | 1(2) |
| Nausea/vomiting | 1(2) | 48(73) | 9(13) |
| Mucositis | 8(12) | 1(2) | – |
| Urinary | 1(2) | – | – |
| Oesophagitis | 4(6) | 1(2) | – |
| Neurological | 8(12) | – | – |
| Auditory | 3(5) | – | – |

accompanying leucopenia. One patient had a mild urinary tract infection. Twelve per cent of patients had a drop in haemoglobin to below $8.0\,g\,dl^{-1}$ while 14% had at least one episode of thrombocytopenia (less than $50\times10^9\,l^{-1}$). Eighty-six per cent of patients experienced grade 3 or 4 vomiting despite prophylactic high dose metaclopramide therapy during treatment with cisplatinum. One patient experienced severe oesophagitis and another severe oral mucositis but otherwise toxicity was generally mild.

The objective toxicity of maintenance therapy was less. Five patients developed leukopenia of $<2\times10^9\,l^{-1}$ on at least one occasion. There were no serious infections. Grade 3 gastrointestinal toxicity occurred in only 18% of cases. Three patients, however, refused maintenace therapy and six withdrew before the six cycles were complete.

The intensity of chemotherapy given during the induction phase to those randomised to each arm of the study was calculated to detect possible imbalance in the groups. Figure 3 shows the percentage of patients who received 80% or more of the ideal scheduled dose for each of the three induction courses. No imbalance was detected. Those randomised to receive maintenance therapy received the same intensity of induction chemotherapy as those randomised to no maintenance.

### Discussion

In this study patients randomised to maintenance therapy had a significantly inferior survival to that of those receiving induction therapy alone. Deaths in the maintenace treated group were predominantly due to uncontrolled small cell cancer and not to toxicity of the maintenance therapy. While other studies have shown limited or no advantage to maintenance chemotherapy, a significant adverse effect has not been seen (Table V).

The median survival of 15.8 months for all treated patients and 25 months for those who achieved complete remission is comparable to that of other groups of patients with limited small cell cancer so treated (Bunn et al., 1987). Those managed with the induction treatment programme alone, however, had a median survival of 19.2 months and 48% were relapse-free at 2 years, which is superior to that generally reported for such patients.

Had maintenance therapy merely failed to improve the results achieved by the initial therapy, it may have indicated that median survivals of 18–20 months are close to the limits of what can be achieved with current conventional chemotherapy and radiotherapy and that prolonging such treatment will not add significantly to the result. The apparent detrimental effect of maintenance therapy is not easily explained on this basis in the absence of evidence of toxicity leading to premature deaths.

The delivery of maintenance chemotherapy may have influenced the amount of chemotherapy given after relapse as relapse therapy was not specified in the protocol. If those who had not received maintenance therapy were able to receive more chemotherapy after relapse than those who had, this may have had a favourable influence on the survival of the non-maintenance group. To examine this possibility, the total drug dose and dose intensity of drug delivery after relapse was assessed in all patients who achieved complete remission. There were no significant differences between the groups. The reason for the significantly inferior survival of those randomised to maintenance therapy is therefore unclear.

Given the small numbers of patients accrued to this study, the absence of a clear explanation for the inferior survival of the maintenance group and the marginal significance of the difference, it is probably wisest to interpret the data as showing no benefit for maintenance chemotherapy. Viewed in this way the results are complementary to those of other larger studies addressing this question (Table V).

Progression outside the irradiated field was the site of primary failure in 49% of cases, indicating that failure of chemotherapy to control metastases remains the major weakness of combined modality therapy. This study provides no encouragement that prolonging treatment with maintenance regimens of the type used will produce

**Figure 3** Percentage of patients receiving >80% of prescribed dose of drug for each course of chemotherapy. CP, Cisplatinum; VP, VP16213; C, cyclophosphamide; V, vincristine; M, methotrexate.

**Table V** Maintenance chemotherapy in small cell lung cancer, randomised studies

| *Author* | *Disease extent* | *Treatment* | | | *Survival* |
|---|---|---|---|---|---|
| Woods *et al.* | LD, ED | Cisplat VP16 x 4<br>+DXRT<br>+PCI | PR<br>CR | CAV x 10<br>zero | overall median 50 wks<br>M *vs* NM, n.s. |
| Bleehen *et al.* | LD, ED | CVM VP16 x 6<br>+DXRT, LD | PR<br>CR | CVM VP16 x 6<br>zero | overall median 57 wks<br>M *vs* NM, n.s. |
| Splinter *et al.* | LD, ED | CA VP16 x 5<br>No DXRT<br>+PCI | NC<br>PR<br>CR | CA VP16 x 7<br>zero | overall median 33–41 wks<br>M *vs* NM, n.s. |
| Harper *et al.* | LD, ED | CV VP16 x 4 | | CV VP16 x 4<br>zero | LD M 48 wks *vs* NM 42 wks n.s.<br>ED M 34 wks *vs* NM 28 wks n.s. |
| Ettinger *et al.* | ED | CAV/HM VP16 x 6–8<br>CAV x 6–8 | CR | Induction<br>zero | CR M 75 wks *vs* NM 61 wks n.s. |
| Einhorn *et al.* | LD | CAV x 6<br>+DXRT | PR<br>CR | Cisplat VP16 x 2<br>zero | PR + CR M 98 wks *vs* NM 67 wks<br>$P=0.009$ |
| Byrne *et al.*<br>(present study) | LD | Cisplat VP16/CVM x 3<br>+DXRT<br>+PCI | NC<br>PR<br>CR | - CVM x 6 | Total M 60 wk *vs* NM 81 wk<br>$P=0.05$ |
| | | Cisplat VP16/CVM x 3<br>+DXRT<br>+PCI | NC<br>PR<br>CM | - zero | |

LD, limited disease; ED, extensive disease; Cisplat, cisplatinum; VP16, VP16213; C, cyclophosphamide; V, vincristine; A, adriamycin; M, methotrexate; H, hexamethylmelamine; DXRT, thoracic irradiation; PCI, prophylactic cranial irradiation.

significant benefit. Other such studies (Table V) have also shown little or no evidence of benefit. It seems unlikely then that protocols relying on increasing the duration of chemotherapy with currently available agents will produce a quantum change in control outside of the irradiated field. Those in which a single combination is used during induction may improve outcome by incorporating a non-cross-resistant regimen as consolidation (Einhorn *et al.*, 1987), but if alternating non-cross-resistant regimens are used during induction no extra benefit accrues with prolonged therapy.

In 12% of our patients the site of first progression was within the irradiated field. This is a somewhat lower figure than reported in trials of similar design (Perez *et al.*, 1984; Perry *et al.*, 1987). As the toxicity of the radiotherapy dose and schedule used was low, the prospect exists for a small improvement in local control by modification of the radiotherapy programme.

Of the patients who achieved complete remission only two relapsed in the central nervous system, implying that prophylactic cranial irradiation as delivered provides sufficient protection against central nervous system relapse. If the overall treatment becomes more successful in the future late central nervous system relapse may become more prominent.

The basic strategy of the induction regimen of alternating cycles of non-cross-resistant chemotherapy with split course radiotherapy and prophylactic cranial irradiation for those in complete remission has produced a complete remission rate of 59% with 48% two-year disease-free survival in those not receiving maintenance therapy. The changes to the dose and scheduling of thoracic radiotherapy may produce an incremental improvement but a significant improvement in the degree of control of metastatic disease will be required to improve long-term outlook. It seems unlikely that maintenance chemotherapy will produce such benefit.

Supported by the Cancer Foundation of Western Australia. We are grateful to Loraine Blunt for expert data management, and to Gail Stewart for typing the manuscript. The cooperation and active support of the following trial participants is acknowledged: Sir Charles Gairdner Hospital – M.J. Byrne, G. van Hazel, F. Cameron, B. Cassidy, P. Leckie, R. Adams, J. Elder, W. Musk, M. Phillips, G. Ryan, P.J. Thompson, A. Tribe, P. Gibson, T. Nicholls; Royal Perth Hospital – M. Leahy, J. Trotter, J. Shepherd; Fremantle Hospital – P. Claringbold.

### References

BLEEHEN, N.M., FAYERS, P.M., GIRLING, D.J. & STEPHENS, R.J. (1986). Controlled trial of maintenance (m) *vs* no maintenance (Nom) chemotherapy of small-cell lung cancer (SCLC). Abstracts IV World Congress on Lung Cancer. *Lung Cancer*, **2**, 118.

BROOKMEYER, R. & CROWLEY, J. (1982). A confidence interval for the median survival time. *Biometrics*, **38**, 29.

BUNN, P.A., LITCHER, A.S., MAKUCH, R.W. and 8 others (1987). Chemotherapy alone or chemotherapy with chest radiation therapy in limited stage small cell lung cancer: a prospective randomised trial. *Ann. Intern. Med.*, **10**, 655.

COX, D.R. (1972). Regression models and life tables. *J. Stat. Soc. Series B*, **34**, 187.

EINHORN, L., GRECO, F.A., COHEN, H. & BIRCH, R. (1987). Late consolidation with Cisplatinum plus VP16 (PVP16) following induction chemotherapy with cyclophosphamide, adriamycin and vincristine (CAV) in limited small cell cancer (SCLC): a Southeastern cancer study group (SECSG) random prospective study. *Proc. Am. Soc. Clin. Oncol.*, **6**, 166.

ETTINGER, D.S., MEHTA, C.R., ABELOFF, M.D., RUCKDESCHEL, J.C. & AISNER, S. (1987). Maintenance chemotherapy versus no maintenance chemotherapy in complete responders following induction chemotherapy in extensive disease (E.D.) small cell lung cancer (SCLC). *Proc. Am. Soc. Clin. Oncol.*, **6**, 175.

HARPER, P.G., SOUHAMI, R.L., ASH, C.M., SPIRO, S.G., TOBIAS, J.T. & GEDDES D. (1987). Treatment duration in small cell lung cancer (SCLC): a randomised comparison of 4 versus 8 courses of initial chemotherapy (I.Ct.) + or − further chemotherapy on relapse. Fourth European Conference on Clinical Oncology and Cancer Nursing (abstract).

KAPLAN, E.L. & MEIER, P. (1958). Nonparametric estimations from incomplete observations. *J. Am. Stat. Assoc.*, **53**, 457.

MILLER, A.B., HOOGSTRATEN, B., STAQUET, M. & WINKLER, A. (1981). Reporting results of cancer treatment. *Cancer*, **47**, 207.

OSTERLIND, K., HANSEN, H.H., HANSEN, H.S., DOMBERNOWSKY, P., HANSEN, M. & RORTH, M. (1986). Chemotherapy versus chemotherapy plus irradiation in limited small cell lung cancer. Results of a controlled trial with 5-years follow-up. *Br. J. Cancer*, **54**, 7.

PEREZ, C.A., EINHORN, L., OLDHAM, R.K. and 14 others (1984). Randomised trial of radiotherapy to the thorax in limited small cell carcinoma of the lung treated with multiagent chemotherapy and elective brain irradiation: a preliminary report. *J. Clin. Oncol.*, **2**, 1200.

PERRY, M.C., EATON, W.L., PROPERT, J.C. and 9 others (1987). Chemotherapy with or without radiation therapy in limited small cell carcinoma of the lung. *N. Engl. J. Med.*, **316**, 912.

PETO, R., PIKE, M.C., ARMITAGE, P. and 7 others (1977). Design and analysis of randomised clinical trials requiring prolonged observation of each patient. II Analysis and examples. *Br. J. Cancer*, **35**, 1.

SPLINTER, T., McVIE, J., DALESIO, O. and 20 others (1986). EORTCO8825: induction versus induction plus maintenance chemotherapy in small cell lung cancer. *Proc. Am. Soc. Clin. Oncol.*, **5**, 188.

WOODS, R.L. & LEVI, J.A. (1984). Chemotherapy for small cell lung cancer (SCLC): a randomised study of maintenance therapy with Cyclophosphamide Adriamycin and Vincristine (CAV) after remission induction with Cis-platinum (CISDDP), VP16213 and radiotherapy. *Proc. Am. Soc. Clin. Oncol.*, **3**, 214.